# AI-driven early diagnosis of specific mental disorders: a comprehensive study

**DOI:** 10.1007/s11571-025-10253-x

**Published:** 2025-05-05

**Authors:** Firuze Damla Eryılmaz Baran, Meric Cetin

**Affiliations:** Department of Computer Engineering, Pamukkale University, 20160 Denizli, Turkey

**Keywords:** Artificial intelligence, Mental disorders, Machine learning, Deep learning, Clinical decision support systems

## Abstract

One of the areas where artificial intelligence (AI) technologies are used is the detection and diagnosis of mental disorders. AI approaches, including machine learning and deep learning models, can identify early signs of bipolar disorder, schizophrenia, autism spectrum disorder, depression, suicidality, and dementia by analyzing speech patterns, behaviors, and physiological data. These approaches increase diagnostic accuracy and enable timely intervention, which is crucial for effective treatment. This paper presents a comprehensive literature review of AI approaches applied to mental disorder detection using various data sources, such as survey, Electroencephalography (EEG) signal, text and image. Applications include predicting anxiety and depression levels in online games, detecting schizophrenia from EEG signals, detecting autism spectrum disorder, analyzing text-based indicators of suicidality and depression, and diagnosing dementia from magnetic resonance imaging images. eXtreme Gradient Boosting (XGBoost), light gradient-boosting machine (LightGBM), random forest (RF), support vector machine (SVM), K-nearest neighbor were designed as machine learning models, and convolutional neural networks (CNN), long short-term memory (LSTM) and gated recurrent unit (GRU) models suitable for the dataset were designed as deep learning models. Data preprocessing techniques such as wavelet transforms, normalization, clustering were used to optimize model performances, and hyperparameter optimization and feature extraction were performed. While the LightGBM technique had the highest performance with 96% accuracy for anxiety and depression prediction, the optimized SVM stood out with 97% accuracy. Autism spectrum disorder classification reached 98% accuracy with XGBoost, RF and LightGBM. The LSTM model achieved a high accuracy of 83% in schizophrenia diagnosis. The GRU model showed the best performance with 93% accuracy in text-based suicide and depression detection. In the detection of dementia, LSTM and GRU models have demonstrated their effectiveness in data analysis by reaching 99% accuracy. The findings of the study highlight the effectiveness of LSTM and GRU for sequential data analysis and their applicability in medical imaging or natural language processing. XGBoost and LightGBM are noted to be highly accurate ML tools for clinical diagnoses. In addition, hyperparameter optimization and advanced data pre-processing approaches are confirmed to significantly improve model performance. The results obtained with this study have revealed the potential to improve clinical decision support systems for mental disorders with AI, facilitating early diagnosis and personalized treatment strategies.

## Introduction

After the world wars, the recognition of behavioral disorders in patients with head trauma and the investigation of the causes of these disorders led to the emergence and advancement of the field of psychiatry. Psychiatry is a type of science that conducts studies to understand the relationship between brain physiology and functions and a person’s behavior. In this field of practice, the outputs of brain activities are observed and explained with measurable behaviors. Mental disorders are basically divided into two branches: clinical and experimental. Clinical applications examine the effects of an existing brain injury or brain disorders on a person’s psychology. Experimental applications examine the psychological processes of normal people without any brain damage or disorders under different and various conditions. Tests developed by psychologists measure patients' behaviors, motor-language skills and their use, memory and attention. In the first studies, psychologists developed tests suitable for interpreting simple motor movements. In later studies, they began to look for tests that provided more concrete data and verified the accuracy of the detection within a certain consistency index. Thanks to the development of technology, computer systems can monitor patients' motor movements and report accordingly, which has increased test reliability and data accuracy. In addition, the use of neuroimaging techniques such as computerized tomography (CT) and magnetic resonance (MR) in brain imaging and detection of damaged areas is a great advantage for mental disorder studies.

Behavioral disorders with biological causes, neurodegenerative diseases such as Alzheimer, which occur due to structural disorders in the nervous system, depression, bipolar disorder, anxiety, attention deficit, learning disability or schizophrenia are examined within the scope of mental disorder research (Eryılmaz Baran 2024). Since these types of disorders can affect cognitive and behavioral functions, when they are not diagnosed correctly, they both endanger the individual’s life and create difficult processes for their environment (Patterson et al. 2024). Despite advances in medical imaging and diagnostic tools, accurate and fast detection of mental disorders is difficult due to overlapping symptoms and some features. However, the accuracy and speed of these detections are critical to increasing the effectiveness of treatment plans. Traditional diagnostic methods often rely on subjective evaluations, which may lead to delayed interventions. With the rapid development of AI technologies, particularly machine learning (ML) and deep learning (DL), there is growing potential to enhance the accuracy, reliability, and efficiency of diagnosing mental disorders (Yasin et al. 2023). Current research on the application of AI to this area is limited. In addition, the optimization of algorithms to maximize performance across diverse datasets is insufficiently explored. The aim of this study is to evaluate the applicability of artificial intelligence methods to different types of mental disorders from a broad perspective, to classify such disorders using these methods, and to examine the effects of hyperparameter optimization on performance in this process. Our study does not aim to make a direct clinical diagnosis. Additional validation studies on big and more diverse datasets are required for clinical adaptations. As artificial intelligence models become more accessible and interpretable for clinicians, the applicability of these models in the health field will increase. In this sense, the types of disorders in which the algorithms reach higher accuracy rates and the effect of optimization processes on the classification success are revealed in detail. It is thought that the obtained results will make significant contributions to improving diagnosis and treatment planning.

## Related work

Mental disorders enclose a wide range of conditions that affect cognitive, emotional, and behavioral functions, posing complex diagnostic and therapeutic challenges. Long-short-term exposure to a severe psychological event or biological changes in brain chemistry can lead to neurological abnormalities. In addition, some individuals may have a genetic predisposition to these disorders. Traditional diagnostic methods often rely on subjective assessments, which leads to inconsistencies and inaccuracies in classification and prognosis. Furthermore, the complex interaction of various factors contributing to these disorders necessitates a multidimensional approach for a comprehensive analysis. In this paper, common mental disorders were examined and ML and DL techniques were used to analyze mental disorders. Data preprocessing, feature extraction and selection, model development, classification, and validation proceses were performed to make predictions. In this section, a comprehensive of literature information is presented about some mental disorders analyzed using artificial intelligence models before presenting the simulation results of this study.

### Schizophrenia disorder

Schizophrenia disorder (SD) is a psychiatric disorder that can be diagnosed with positive symptoms such as delusions, shallowness in emotions and thoughts and inability to convey them, negative symptoms such as sociophobic behaviors and decreased self-expression and conversation, and cognitive symptoms such as impairment in attention, memory, and executive functions (Summakoğlu and Ertuğrul 2018). Many factors, such as genetic factors, morphological chemical and physiological changes in the brain, and endocrine factors may be effective in the emergence of schizophrenia. According to research data, there is a shrinkage in the frontal lobe, a disorder in the thalamus’s function of filtering and sorting perceptions, and a disorder in the circuits between the prefrontal cortex and the cerebellum in patients with schizophrenia. Past research has shown that people diagnosed with schizophrenia have a deficiency in input regulation when stimuli reach the brain. When too much information is transmitted to the brain, the patient cannot sort and integrate this information for adaptation purposes. Although the first symptoms of schizophrenia are based on observation, they can be easily detected through imaging when the flow of stimulation in the relevant areas of the brain is interrupted and the stimulation intensifies. In general, although symptoms can be visually analyzed in the diagnosis of diseases, biological tests that will provide direct confirmation are limited. Especially with the monitoring of neurological factors and MRI techniques, symptoms are tried to be interpreted correctly and the diagnosis process is carried out accordingly. There are many studies in the literature that perform SD classification and diagnosis using artificial intelligence models.

In a study that proposed a machine learning method based on handwriting data for the diagnosis of schizophrenia and bipolar disorder, an alternative to costly methods such as MRI was presented (Javeed et al. 2025). The proposed model eliminates data imbalance by determining statistically significant handwriting features and achieves high success with 96% accuracy using artificial neural networks. In Yan et al. (2019), a multi-scale recurrent neural network (RNN) model using functional MRI (fMRI) time series was designed for schizophrenia classification and 83.2% accuracy was achieved. In Qureshi et al. (2019), brain MRI data were analyzed using a three-dimensional CNN to distinguish schizophrenia patients from healthy individuals and 98.09% accuracy was achieved. In the neurodevelopmental model of schizophrenia, minor physical anomalies are considered neurodevelopmental markers of schizophrenia. In Jeng et al. (2024), classical statistical model, logistic regression (LR), decision tree (DT) and RF models were used to predict the risk of schizophrenia based on these anomalies. The results showed that especially the RF model had the highest accuracy in predicting schizophrenia and physical anomalies such as skull height, mouth width, high palate, and frowning tongue were associated with the risk of schizophrenia. In a study evaluating whether ML techniques could help in the diagnosis of schizophrenia, it was aimed to support the diagnosis of schizophrenia based on resting state EEG data (De Miras et al. 2023). By using features that provide better discrimination on resting state EEG data in machine learning algorithms such as KNN, LR, DT, RF and SVM, schizophrenia patients could be distinguished from healthy individuals with high accuracy. In particular, SVM achieved the highest classification feature (AUC = 0.89). In Soria et al. (2023), a ML method based on the XGBoost algorithm was proposed to analyze schizophrenia patients with EEG signals. According to the results, the XGBoost-based method outperformed other supervised machine learning methods with an accuracy value of 94%. Bagherzadeh et al. (2022) presented a hybrid deep learning framework for schizophrenia detection in multi-channel EEG signals. Using data obtained from EEG signals and CNN-LSTM models, healthy individuals and schizophrenia patients could be distinguished with high accuracy (99.90%). In Shoeibi et al. (2021), various deep learning-based methods have been proposed for automatic schizophrenia diagnosis using EEG signals and these methods have been compared with conventional ML methods. The results show that the CNN-LSTM model performs best with an accuracy rate of 99.25%, outperforming previous studies in diagnosing schizophrenia. In the study of Aslan and Akin (2022), features were automatically extracted from EEG signals for the detection of schizophrenia. EEG signals were converted to 2D with continuous wave transform (CWT) and classification was performed with 98–99.5% accuracy using the Visual Geometry Group 16 (VGG16) network. Grover et al. (2023) developed the EEG-based Schizo-Net model to detect biomarkers of schizophrenia with 99.84% accuracy. The model performance is superior to existing methods with a multimodal deep learning architecture trained after preprocessing.

### Depression and anxiety disorder

Depression can occur due to environmental factors (such as exposure to intense stress, substance abuse, serious changes in the individual’s life), genetic factors, or combination of all these factors. Depression is not determined by factors such as a specific age range, gender, or socioeconomic status. Symptoms of the disease include psychological changes such as loss of self-esteem and loss of joy in life. During periods when the severity of the illness increases, such as major depression, individuals may also experience suicidal ideation and suicide attempts (American Psychiatric Association 2013). Diagnosis of depression is based on subjective methods such as face-to-face interviews between the doctor and the patient and psychometric questionnaires. In the study conducted by Mumtaz et al. (2017), it was concluded that depression can be diagnosed by examining EEG signals and interhemispheric asymmetry in the signals of some regions in the brain. The restlessness experienced by the individual as a result of feeling that their physical and spiritual existence is in danger causes fear and anxiety. This state of restlessness and anxiety experienced by the individual, even in the absence of a causeless and concrete danger, is called anxiety disorder (AD). An individual with AD sees the risk of the situation in which they are under threat as high, but their coping skills are low. For disorders such as depression and anxiety, diagnosis is made with the support of medical imaging techniques after the emergence of symptoms based on observation in the initial stage. According to the diagnostic and statistical manual of mental disorders (American Psychiatric Association 2013), major depression criteria are decreased desire and interest, depressed mood, decreased or increased sleep and appetite, psychomotor slowdown, guilt, difficulty in concentration, decreased energy, and suicidal ideation. There are many studies in the literature that classify and diagnose depression and AD using artificial intelligence models.

Richter et al. (2024) compared cognitive biases such as sselective and spatial attention, anticipation, interpretation, memory, and cognitive control biases to predict anxiety and depression symptoms. The study found that interpretation and anticipation biases, in particular, had the strongest associations with symptom severity. In the study conducted by Yasin et al. (2024a), the interaction between physical disability and mental health in the detection of depression recurrence specific to quadriplegic patients was investigated. The dataset included clinical and non-clinical data including EEG signals, patient-reported outcomes, and behavioral indicators. As a result of the experiments conducted, the proposed approach reached 98% accuracy rate and logistic regression was determined as the most effective classification method. Thus, an important contribution was made to understanding the relationship between physical disability and mental health. A study by Spinrad et al. (2024) examined the effect of reviewing action suggestions given by therapists in therapy sessions on depression and anxiety symptoms. The generalized estimating equations model used evaluates the relationship between the measurement and changes in clients’ depression and anxiety scores. The model achieved 76% accuracy in capturing action suggestions and 71.1% accuracy in reviewing them. The results showed that, particularly in patients with mild depression, more frequent review of action suggestions by therapists was associated with a significant improvement in depression symptoms. In Feng et al. (2025), a deep learning model was developed and validated to predict depression and anxiety in patients with type 2 diabetes. Using regional electronic health records data, the model robustly predicted the onset of depression and outperformed other baseline models, which offers significant potential for clinical use. Aldayel and Al-Nafjan (2024) studied EEG-based anxiety detection, comparing different labeling, feature extraction, and classification algorithms. Using Hamilton anxiety rating scale (HAM-A) for labeling and DWT for feature extraction, the RF classifier achieved the highest accuracy of 87.5%, outperforming other methods like AdaBoosted Bagging (79%). In Lee et al. (2024), time series step count and sleep data obtained from wrist-worn activity trackers were used to diagnose geriatric depression and anxiety. The basic structure of the proposed DL model was designed to perform multi-label classification for depression and anxiety by processing mixed input data. Various DL models, including CNN and LSTM were applied to process time series data, and model selection was made by comparing the performances of hyperparameters. Significant results were obtained in multi-label classification of depression and anxiety with a Hamming loss score of 0.0946 in ResNet. In a study using the anxiety gamers dataset (Latubessy et al. 2024), the weighted Naive Bayes model, Gaussian Naive Bayes, multinomial Naive Bayes and categorical Naive Bayes models were analyzed. As a result of the comparison, the weighted Naive Bayes model performed best with an accuracy rate of 99.22%. In another study (Gharpure and Gharpure 2022), C-support vector machine (SVC) and multi-layer perceptron (MLP) models were used on the anxiety gamers dataset to investigate the anxiety effect of games on gamers. The study investigated the relationship between factors such as game type, playing time, age, gender and stress levels. MLP showed a higher result with an accuracy rate of 0.881 than SVC’s accuracy rate of 0.878. In another study by Yasin et al. (2024b), experiments were conducted using the healthy brain network (HBN) database consisting of 100 patients, firstly EEG signals were denoised and 16,383 features were extracted. Then, feature selection was performed and analyses were performed with classifiers such as SVM, DT, NB and LR. The results revealed the effects of depression on memory and learning capacity by reaching 100% accuracy rate with the decision tree classifier.

### Autism spectrum disorder (ASD)

Autism spectrum disorder (ASD) is a disorder that occurs due to differences in the structural functioning of the brain due to the effects of genetic and environmental factors. In the diagnosis of autism, more than one symptom should emerge and these should be observed systematically. In terms of diagnosis, not only is a specific action and behavior monitored, but also whether the movements are in a certain sequence or frequency or how they are reacted to which event is analyzed. The individual’s behavioral patterns are one of the most important factors in diagnosing ASD. According to the DSM-IV-TR diagnostic manual, 6 symptoms and above are sufficient to diagnose autism (First et al. 2004) Dinçer. These symptoms include content-based disorders at the level of social communication and interaction, repetitive and limited behaviors. Symptoms include lack of social and emotional reciprocity, stereotypical and repetitive use of language or idiosyncratic (personal) language use, and persistent interest in parts of objects. Various studies have also shown that various motor disorders such as muscle weakness (hypotonia), gross motor dysfunction (apraxia) and walking on tiptoes are encountered in ASD (Huda et al. 2024). In particular, if there is an effective flexibility and decrease in social behaviors observed in similar age groups, the diagnostic process should begin. Only in this way can it be determined whether the behaviors are a normal sociological action or whether they are due to a neuropsychological cause. There are many studies in the literature for the classification and diagnosis of ASD using artificial intelligence models.

In one of the studies in the literature, a model that can diagnose attention deficit hyperactivity disorder (ADHD) and autism was developed (Sen et al. 2018). In this study, 64.3% classification accuracy was achieved using linear SVM by combining multiple features obtained from MRI and fMRI images. Guo et al. developed a deep neural network (DNN) and a novel feature selection method to distinguish patients with ASD from typically developing individuals (Guo et al. 2017). The feature selection method showed the best classification accuracy performance of 86.36% by selecting features with high discriminative power from trained sparse auto-encoders, helping the DNN generate low-dimensional and high-quality brain functional connectivity patterns. In Parvathy et al. (2025), a novel DL model named ViT-ARDNet-LSTM was presented to classify ASD based on MRI images by integrating adaptive residual densenets and LSTM. The proposed model achieved better diagnostic performance compared to traditional methods by addressing issues such as MRI image variability and feature extraction, and reached 94% accuracy rate. In Dekhil et al. (2018), a probabilistic SVM classifier was designed for ASD using power spectral densities as input features. With this model, approximately 90% success was achieved in sensitivity, specificity and accuracy. Wang et al. designed a SVM-recursive feature elimination algorithm (Wang et al. 2019) using resting-state functional magnetic resonance imaging data, and then extracted high-level features with a sparse auto-encoder with two hidden layers and achieved a classification accuracy of 93.59%. In Lakhan et al. (2023), a Federated Learning CNN-LSTM (FCNN-LSTM) model using multimodal datasets for ASD detection was proposed. Furthermore, real-time ASD IoT applications were designed based on a trained dataset, where ASD patients demonstrated their efficiency and learning abilities. Simulation results show that the proposed frameworks achieve an ASD detection accuracy of approximately 99% compared to all existing ASD frameworks. In Raj and Masood (2020), Naive Bayes, SVM, LR, KNN, neural network and CNN were used to predict and analyze ASD problems in children, adolescents and adults. According to the results, CNN-based prediction models achieved 99.53%, 98.30%, 96.88% accuracy in diagnosing ASD in adults, children and adolescents, respectively. In Asif Mohamed et al. (2022), convolutional neural networks with transfer learning and flask framework were designed. In this study, Xception model was compared with other deep learning models previously studied with this dataset. Xception model gave results with 0.93 accuracy rate.

### Dementia and Alzheimer

Dementia is a type of disease in which there is a disorder in human memory and similar mental abilities. Dementia can occur in one or more of the mental abilities. For example, the patient may experience symptoms such as impaired thinking ability, difficulty in making decisions, speech disorders and memory disorders. The most common causes of dementia are communication disorders between brain nerves and death of brain cells. Factors that may increase the risk of dementia include addictive substance use, diabetes, high blood pressure or cholesterol, and weight problems. The combination of these disorders or hereditary factors can trigger dementia. Traditional approaches to identifying dementia rely primarily on clinical examinations, analysis of medical records, and administration of cognitive and mental tests (Javeed et al. 2024). The mini-mental state examination is applied to patients for dementia diagnosis (Dick et al. 1984). If the test results suggest dementia, patients undergo more comprehensive tests. These tests include psychometric and neurological evaluation, physical examination, and taking the patient’s history. After the mini-mental state examination, medical imaging techniques, EEG, spinal fluid collection procedures and biochemical tests can be applied. Although it is often mentioned together with Alzheimer in practice, Alzheimer is a type of dementia but is not the same disease. A person with Alzheimer disease may experience some cognitive disorders and especially memory loss. The main reason for using imaging techniques for diagnosis in Alzheimer is that the disease gives symptoms based on observation and/or behavioral tests. A definite and clear diagnosis is made by interpreting imaging techniques. In Alzheimer patients who show symptoms in the early stages, the effects and progression of the disease can be reduced with the treatment process. There are many studies in the literature on classifying and diagnosing dementia and Alzheimer diseases using artificial intelligence models.

In Javeed et al. (2024), a hybrid system based on statistical and ML methods using patients’ electronic health records for early prediction of dementia was developed. An ensemble voting classifier based on five different ML models (DT, Naive Bayes, LR, SVM and RF) was designed for classification. A cross-validation approach was used to evaluate the performance of the proposed diagnostic system to solve the overfitting problem of the ML models. According to the experimental results, the proposed diagnostic method achieved an accuracy rate of 98.25%. Nyholm et al. investigated whether ML algorithms can predict dementia and which sleep disturbance factors affect dementia (Nyholm et al. 2024). The study used five ML algorithms (gradient boosting, LR, Naive Bayes, RF and SVM) and data on the elderly population in Sweden. The results showed that there is an association between sleep disturbances and dementia and that machine learning algorithms can successfully predict this risk. The gradient boosting method achieved the best performance with a 92.9% accuracy rate. In Zhang et al. (2024), a multi-factorial XGBoost model was constructed to predict whether patients with different types of dementia would survive 1, 3, 5, or 10 years. The models predicted the risk of death of dementia patients with high accuracy (82%) using a small number of clinical features. Thus, early intervention and personalized treatment strategies can be applied in clinical practice. Gajjar et al. (2024) explored using Generative Adversarial Networks (GANs), variational autoencoders, and diffusion models for generating synthetic MRI data related to Alzheimer’s and Parkinson’s diseases. DenseNet outperformed ResNet in disease detection, achieving 80.84% accuracy for Alzheimer’s and 92.42% for Parkinson’s when trained on diffusion-generated images. In Kaya and Çetin-Kaya (2024), a DL model that optimizes hyperparameters was developed to automatically classify the severity of dementia from MRI data. The proposed model achieved 99.53% accuracy by calculating hyperparameters with particle swarm optimization (PSO). In Deshpande et al. (2024), a hybrid model combining CNN and LSTM networks was developed to predict the progression of Alzheimer disease. PSO was used to optimize the model parameters. In the study, PSO + CNN–LSTM and CNN–LSTM were compared separately. The PSO + CNN–LSTM hybrid model gave the highest success with an accuracy rate of 98.13%.

### Attention deficit hyperactivity disorder

ADHD is a mental disorder that negatively affects the individual’s social, professional and daily life due to symptoms such as attention deficit and hyperactivity. Although it was previously considered that the disorder was related to the person’s behavior, it was later determined that the cause of the disease was based on a neurodevelopmental factor. ADHD is especially seen in childhood and adolescence, but it can rarely occur later in adults. It is possible to divide ADHD diagnostic methods and tools into two main categories. These are; inattention and impulsivity. Inattention can be easily observed in the individual’s daily behavior and movements. The clearest and most basic indicators of inattention are the person’s simple mistakes and not being able to focus on an event for a long time. In hyperactivity, or impulsivity, the person often plays with their hands or feet, while children often run around or exhibit behaviors such as climbing. This situation is seen as a subjective feeling of restlessness in young people or adults. In addition to observable symptoms, it is also possible to diagnose the disease with mental disorder diagnostic methods. Among the neuropsychological findings, observation of excessive motor activity, the presence of time processing disorders, dislike of delaying gratification, and determinations of desire and dysfunction in striving towards a goal are important diagnostic tools for the detection of individuals with ADHD (Loh et al. 2024). Neuroimaging technologies, which are generally used together with ML algorithms, are also being investigated as biomarkers in the diagnosis of this disorder. In children with ADHD, there is a general decrease in volume in certain brain regions (particularly a proportionally greater decrease in volume in the prefrontal cortex on the left side). In comparisons made with the control group in individuals with ADHD, there is thinning in the posterior parietal cortex. Other brain structures in the prefrontal–striatal–cerebellar and prefrontal–striatal–thalamic circuits have also been found to differ between individuals with and without ADHD. The brain structure of people with suspected disease or showing symptoms is examined by taking into account the MRI results and as a result of the evaluations here, ADHD is diagnosed. The region that is suppressed due to the change in the brain structure of the individual is taken into account in the classification according to the nature of the symptoms. There are many studies in the literature that perform ADHD classification and diagnosis using artificial intelligence models.

A study investigated EEG decomposition techniques using independent component analysis (ICA) and robust decomposition methods (STFT and DCT) to improve ADHD detection (Deshmukh and Khemchandani 2025). The results indicated that the STFT method outperformed DCT, with the highest accuracy achieved by STFT-XGBoost and Random Forest classifiers, offering potential for automatic, early ADHD diagnosis and personalized treatment. In Alsharif et al. (2024), DT, RF, SVM and MLP models were designed using both data collected from ADHD patients and data from a control group of people without ADHD. According to the results obtained, SVM achieved a high accuracy rate of 91%. The accuracy rates of MLP, RF and DT were calculated as 89%, 87% and 78%, respectively. In another study (Yoo et al. 2024), a ML model using eye-tracking data was proposed for ADHD diagnosis. Eye-tracking data were collected using a digital device during the performance of five behavioral tasks measuring selective attention, working memory, and response inhibition. The proposed model showed a high accuracy of 76.3% in identifying ADHD. In Saurabh and Gupta (2024), a deep learning-based model was developed for ADHD classification by analyzing the functional connectivity obtained from resting-state fMRI data. The modified DL-based bidirectional long short-term memory model, which automates the classification of ADHD, achieved successful results in ADHD classification with 87.5% accuracy rate. In a different study (Ghasemi et al. 2022), a machine learning model using event-related potentials (ERP) data was developed to accurately distinguish between ADHD patients and healthy controls. By combining complementary features and using various ML algorithms, the model achieved an average accuracy of 99.85%. Kasim used neighborhood component analysis to select the most effective features by extracting features with multitaper and multivariate variational mode decomposition methods for the detection of ADHD from EEG signals (Kasim 2023). The deep learning model trained with the selected features successfully distinguished ADHD patients from the control group with 95.54% accuracy rate, outperforming the conventional methods (LDA: 76.38%, SVM: 81.69%).

### Bipolar disorder

Bipolar disorder (BD) is a mood disorder that can be observed as manic and depressive periods and is increasing in frequency in society. Apart from manic and depressive periods, there are also cases where the person goes into a normal mood. It is thought that 60–80% of the risk of developing the disease is due to genetic factors (Craddock and Sklar 2013). In addition, if the individual has had a stroke, traumatic injury, or diseases such as multiple sclerosis (MS), it is possible for bipolar disorder to occur due to neurological causes. The behaviors exhibited by people with BD may resemble the symptoms of other neurological diseases. In this case, medical imaging techniques can be used to distinguish whether the person has a migraine or is in BD. Clinicians use the DSM-5 criteria to diagnose bipolar disorder (American Psychiatric Association 2013). Bipolar disorder is examined in two main categories: type 1 and type 2. According to DSM-5, the presence of mania, hypomania and depression periods at certain periods supports the diagnosis. For bipolar diagnosis, comprehensive psychiatric evaluations are used to examine the patient’s mood and behavior. The patient’s past mood swings, depression, mania or hypomania episodes are evaluated. A family history of bipolar disorder or other mental illnesses is questioned. Genetic predisposition may play an important role in this disorder. Different scales (such as the young mania rating scale or the beck depression inventory) can be used to better understand the symptoms of the disorder (American Psychiatric Association 2013). In addition, medical tests such as blood tests or brain imaging may sometimes be performed to rule out other physical conditions (such as thyroid disorders) that may cause symptoms similar to bipolar disorder. There are many studies in the literature on the classification and diagnosis of BD using artificial intelligence models.

According to the test results of a study aiming to distinguish BD and MDD using SVM, 19 out of 26 BD patients and 20 out of 26 major depressive disorder (MDD) patients were identified with 75% accuracy (Rubin-Falcone et al. 2018). According to the results, further investigation of regional gray matter volume was recommended to predict BD before a manic or hypomanic episode occurs. It is difficult to distinguish bipolar disorder and major depressive disorder based on depressive symptoms. In Gao et al. (2017), it was proven that independent components extracted from fMRI data carry discriminative information and can be used for classification. As a result of the classification performed with support vector machines, 5 fMRI components were determined that could distinguish BD and MDD with 93% accuracy and these components were found to be concentrated in regions such as the prefrontal cortex, default mode network and thalamus etc. In Uyulan et al. (2021), an EEG-based diagnostic model for MDD was developed using deep learning approaches with ResNet-50, MobileNet and Inception-v3 frameworks. According to the frequency-based classification results, the delta frequency reached the highest accuracy (90.22% with ResNet-50) compared to other frequencies. As a result of the tests conducted on the left and right brain, the right brain had a higher accuracy rate. The highest accuracy rate for the right brain was MobileNet with 92.66%, while the highest accuracy rate for the left brain was again MobileNet with 89.33%. Mikolas et al. (2024) used SVM to early diagnose and classify individuals at risk of bipolar disorder based on brain structural features such as cortical thickness. The analyses concluded that brain structural changes were detected in individuals at risk of BD and that this machine learning method could help in early diagnosis of the disease. The best performance was obtained with the BPSS-P scale with 63.1% balanced accuracy. In Metin et al. (2024), bipolar disorder diagnosis was performed with two DL methods (1D-CNN and 2D-CNN combined with LSTM) using EEG signals. According to the results, the 2D-CNN method successfully distinguished BD patients from the control group with an accuracy rate of 95.91%, and the 1D-CNN + LSTM method with an accuracy rate of 93%. It was also stated that electrode activities in the prefrontal regions (F4, C3, F7 and F8) produced dominant features to detect the BD class. Karthik and Sudha (2021) proposed a rank-based gene biomarker identification and classification framework to identify overlapping and non-overlapping gene patterns of BD and SD. As a result of this experiment, seven biomarkers were identified as overlapping genes. Overlapping genes are eliminated to increase the diagnostic accuracy of disorders. The performance of the proposed system was evaluated with standard existing ML algorithms. The proposed model achieved 97.01% accuracy in BD and 95.65% in SD, indicating that genetic biomarkers play an important role in disease diagnosis. In a different study, a personalized dosage model based on ML and DL techniques was developed for valproic acid (VPA) treatment in patients with bipolar disorder (Zheng et al. 2022). Among the nine compared models, namely XGBoost, LightGBM, CatBoost, RF, GBDT, SVM, LR, ANN, and TabNet, CatBoost was selected to establish the best performing personalized medication model (accuracy = 0.85, AUC = 0.91). As a result, the personalized VPA medication model based on CatBoost for patients with BD was reported to have good predictive ability, providing guidance to clinicians in recommending the most appropriate medication regimen. In Allayla and Ayvaz (2024), a new methodology based on big data architecture is proposed for early detection of health condition risks, prevention and reduction of the number of suicidal thoughts. After pre-processing the raw data, it was classified using Naive Bayes, LR, linear SVC, DT, RF and MLP. In addition, all models were compared with 4 different feature extraction methods. The MLP model achieved 93.47% accuracy with the Unigram + Bigram + CVC-IDF feature extraction method. In Eswar et al. (2023), a “transformation block” that provides privacy and robustness to detect depressive symptoms in texts shared on online social media platforms has been developed. The transformation block allows DL models to identify linguistic markers of depression and provides a significant improvement in the performance of depression detection. The transformation block has been included in BERT, Bi-LSTM, LSTM, stacked LSTM and the proposed Fourier transform depression detection (FTDD) deep learning models and the results have been compared. The most successful accuracy rate is the proposed FTDD model with a rate of 0.95. Gupta and Pirzada (2023) proposed a model to help detect depression from social media posts. Combining global vectors for word representation and LSTM networks, this model achieved 93% accuracy with just 30 epochs.

## Methods

### Artificial intelligence

The first AI systems, founded by Alan Turing and John McCarthy, focused on rule-based approaches and symbolic reasoning. Despite the limitations in computational load and data availability during this period, the emergence of machine learning algorithms in the late twentieth century revolutionized the field of AI. Methods such as neural networks, genetic algorithms, and Bayesian networks have enabled systems to learn from data and improve their performance over time. Advances in algorithms, combined with advances in hardware, particularly GPUs, have rapidly advanced AI capabilities. The twenty-first century has witnessed the rise of deep learning, a branch of machine learning that uses multilayered neural networks to extract high-level features from data. Deep learning has taken AI applications to new heights, enabling significant advances in image recognition, natural language processing, and other complex tasks. In recent years, artificial intelligence methods provide innovative solutions to the medical world by providing revolutionary advances in early diagnosis, disease classification and personalized treatment approaches in the field of health. With the ability to analyze huge amounts of data and uncover complex patterns, AI has the potential to enhance clinical decision-making and improve outcomes. AI-driven systems facilitate early diagnosis of diseases such as cancer, cardiovascular disease, and neurological disorders by detecting abnormalities in X-rays, MRIs, and CT scans. They can also predict disease progression and treatment response based on imaging data, allowing for personalized treatment plans for patients.

**Fig. 1:**
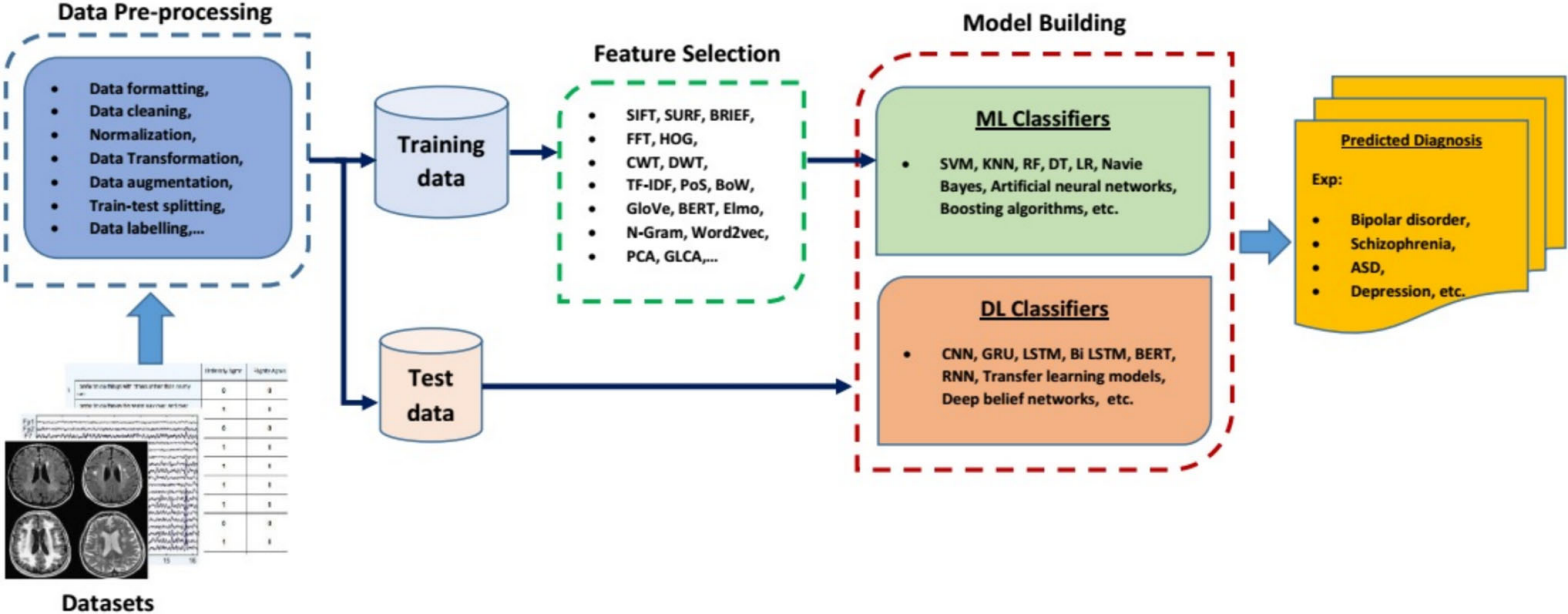
A general flow chart of the data processing pipeline of machine learning and deep learning models

Machine learning applications, which use three different learning methods such as supervised, unsupervised and reinforcement learning to solve issues, focus on the development of models that allow computers to learn from data without being explicitly programmed. Supervised learning algorithms try to find the most optimal function to match the input features of labeled training data with the output data and make the best prediction. This method is mostly used for regression and classification problems. Unlike supervised learning, unsupervised learning tries to find patterns or structures in unlabeled data. Unsupervised learning methods are preferred in applications such as clustering, dimensionality reduction, and anomaly detection. Reinforcement learning is a machine learning method inspired by behavioral psychology, where an agent learns to interact with its environment to achieve a goal (cumulative reward). Reinforcement learning has applications in a wide range of fields, including robotics, autonomous vehicles, recommender systems, finance, and healthcare. Inspired by the structure and function of the human brain, deep learning models automatically learn hierarchical representations of data through multiple layers of artificial neural networks. This allows them to capture complex patterns and features in input data without the need for feature engineering. Thanks to their ability to learn complex patterns, they can be applied to tasks such as classification, regression, clustering, generative modeling, and reinforcement learning for big and high-dimensional datasets including images, text, audio, and video. DL models trained on big datasets can be fine-tuned using transfer learning or adapted to new tasks with smaller datasets. A general flow chart showing the data processing pipeline of the machine learning and deep learning models used in this study is presented in Fig. 1. The following subsections describe the ML methods and DL approaches used in this paper.

### Machine learning algorithms for diagnosis

Machine learning algorithms aim to find a learning function that best maps input variables to output variables, each suitable for different tasks and data types (Cetin et al. 2024). In the literature, there are parametric learning models such as LR, LDA and perceptron that summarize data with a fixed-size parameter set. Although it is easier to interpret the results and learn the data in parametric methods, these algorithms have limitations such as restrictions, limited complexity and poor fit (Al-Jarrah et al. 2015). Nonparametric learning algorithms are free to learn any mapping function from the training data without making any assumptions. Well-known nonparametric methods include decision trees such as classification and regression trees, and C4.5, Naive Bayes, SVM, neural networks. In addition to the advantages of nonparametric learning algorithms such as flexibility and higher performance, they also have limitations such as requiring much more training data and slower algorithms due to the need for more parameters to be trained. The choice of algorithm depends on factors such as the nature of the data, the complexity of the problem, the desired interpretability of the model, and its performance. This section introduces the machine learning algorithms used in many scientific studies and preferred for the classification process in this paper.

#### eXtreme gradient boosting (XGBoost)

XGBoost is a powerful and popular ML algorithm (Chen and Guestrin 2016) known for its performance and scalability, especially on structured data and regression/classification tasks. It belongs to the category of ensemble learning and is based on the gradient boosting framework. XGBoost extends the gradient boosting algorithm, which combines multiple weak predictions to form a strong prediction model. In gradient boosting, each new tree is trained to correct the errors made by the current tree population. XGBoost trains a set of DTs, where each subsequent tree focuses on capturing the errors of the previous population. This iterative process continues until a certain number of trees are reached or a stopping criterion is met. Each tree is usually shallow (low depth) to prevent overfitting and improve generalization performance. XGBoost optimizes a differentiable loss function (e.g., mean square error for regression, log loss for classification) using gradient descent. XGBoost is also useful in terms of speed and scalability. It supports parallelization of tree structure on multiple CPU cores and can efficiently process big datasets containing millions of samples and features. Important parameters for XGBoost are the number of trees, tree depth, row ratio, learning coefficient and column subsampling. Increasing the parameter of the number of trees improves model performance but may also lead to overfitting problem. The tree depth parameter controls the maximum depth of trees in the model. As this value is increased, the model becomes more complex and an overfitting problem may occur. On the other hand, decreasing the value simplifies the model, which may cause insufficient learning. The row ratio parameter controls the observation ratio of each tree in the model. Keeping the value small reduces the complexity and prevents overfitting. Learning coefficient controls the step interval that updates the optimizer’s weights. If this value is kept small, slower but more accurate updates are obtained. The column subsampling parameter controls the ratio of features used for each tree. As the value decreases, model complexity decreases and overfitting is prevented.

#### Light gradient boosting machine (LightGBM)

LightGBM is a high-performance gradient boosting method developed by Microsoft. It is designed for efficient training of large-scale datasets and is known for its speed, memory efficiency, and accuracy. In LightGBM, each new tree trains a set of trees additively, focusing on capturing the errors of the existing population. One of the key features of LightGBM is its leaf-based growth strategy to create DT. By choosing the leaf with the maximum delta loss (improvement in the loss function) to grow, it allows it to find the best split more efficiently and reduce the number of nodes in the trees. LightGBM uses gradient-based one-side sampling (GOSS) technique to speed up the training process. GOSS randomly downsamples the samples with small gradients while preserving the samples with large gradients (which contribute significantly to the loss function). This approach helps reduce the computational cost of training without compromising the accuracy of the model. LightGBM uses histogram-based algorithms to find optimal splitting points when splitting continuous features into discrete features. It provides faster training and better accuracy by using techniques such as categorical splitting and optimal splitting for categorical features. It supports parallelization of tree structure and GPU acceleration, allowing efficient management of large-scale datasets with millions of examples and features (Ke et al. 2017).

#### K-nearest neighbor

The KNN algorithm is a simple yet powerful ML algorithm (Li et al. 2010) used for both classification and regression tasks. It is a non-parametric, example-based learning algorithm that does not make explicit assumptions about the data distribution, instead relying on the entire training dataset during inference. The basic idea behind this algorithm is to classify or predict the label of a new data point based on the majority vote or the average of its k-nearest neighbors in the feature space. When making predictions for a new data point, the algorithm calculates the distance between the new point and every point in the training dataset. A distance measure such as Euclidean distance, Manhattan distance, or cosine similarity is usually used to determine the closeness of data points. The Euclidean distance is the most common choice and measures the straight-line distance between two points in a multidimensional space. It identifies the k-nearest neighbors based on these distances. For classification tasks, the algorithm assigns the majority class label to the k-nearest neighbors of the new data point. If k = 1, the algorithm assigns the label of the single nearest neighbor. For regression tasks, the algorithm calculates the mean or weighted average of the target values of the k-nearest neighbors and assigns this value as the estimate for the new data point. The choice of the number of neighbors for the parameter k is a very important hyperparameter in KNN. A smaller value of k leads to soft decision boundaries and potentially higher variance, while a larger value of k leads to softer decision boundaries and potentially higher bias. In some cases, it is important to normalize or scale the features before applying the KNN algorithm to ensure that all features contribute equally to the distance calculations. Despite its simplicity, the KNN algorithm can be surprisingly effective, especially in low-dimensional spaces and when the decision boundary is highly irregular (Li et al. 2010). It is a versatile algorithm that is often used as a baseline or benchmark for comparison with more complex models.

#### Random forest

RF algorithm is a powerful ensemble learning method (Cutler et al. 2012) used for both classification and regression tasks due to its robustness, scalability, and ability to handle high-dimensional data with complex relationships. The DT is the basic building block of RFs. RFs use bootstrap sampling to create multiple training datasets of the same size as the original dataset. Each DT in the ensemble is trained on a random subset of the training data (bootstrap sample) and a random subset of the features (feature subsampling). This causes diversity among the trees in the ensemble. In addition to bootstrap sampling, RFs also use feature subsampling. In each split in the DT, only a random subset of features is considered for the split. This randomness helps to uncorrelate the trees in the ensemble and improve generalization performance. RFs combine the predictions of all DTs in the ensemble. For classification tasks, the ensemble prediction is usually determined by majority vote (most frequent class label), while for regression tasks, the individual tree predictions are usually averaged. The randomness introduced during training in RFs helps to prevent overfitting. RFs have several hyperparameters that can be tuned to optimize performance, including the number of trees in the ensemble, the maximum depth of the trees, the minimum number of samples required to split a node, and the maximum number of features to consider.

#### Support vector machines

SVM is a powerful supervised learning algorithm (Mohri 2018) used for classification, regression, and outlier detection tasks, and is widely used in a variety of applications including text classification, image recognition, bioinformatics, and financial forecasting. Especially, SVMs are suitable for high-dimensional problems where the number of dimensions exceeds the number of examples. In its simplest form, SVM creates a hyperplane that best separates data points belonging to different classes in the feature space. This hyperplane is chosen to maximize the margin, which is the distance between the hyperplane and the closest support vectors from each class. Support vectors are the data points that are closest to the decision boundary. These are the critical data points that define the margin and affect the location and orientation of the decision boundary. SVMs use a parameter (C) to control the trade-off between maximizing the margin and minimizing the classification errors. A smaller value of C results in a wider margin but allows more classification errors; a larger value of C results in a narrower margin but less classification errors. The kernel allows SVMs to implicitly map input features to high-dimensional spaces without explicitly computing the transformed feature vectors. This allows SVMs to efficiently handle nonlinear decision boundaries and avoid the computational burden of working directly on high-dimensional spaces.

### Advanced deep learning algorithms for diagnosis

Deep learning models, which offer effective solutions in many application areas such as computer vision, natural language processing, speech recognition and visual object recognition, are artificial neural network-based methods that aim to learn more complex and multi-layered representations of data (Kök and Ozdemir 2020). In the literature, different models such as multilayer neural networks, CNNs and LSTM networks are generally used to obtain effective results on big datasets. Deep learning models have more complex structure than conventional ML methods. Therefore, more parameters or layers are dealt with. In addition, learning abstract representations from data requires powerful computational resources. Difficulties such as high computational costs and overfitting in DL models can be overcome by correctly structuring the model and carefully managing the training process. DL models consist of multiple layers, each with its own function in processing input data and producing the desired output. Some common types of layers found in DL architectures are: input layer, hidden layers, convolutional layers, pooling layers, recurrent layers, normalization layers, dropout layers, fully connected layers and output layer. Additional layers and variations can also be used to improve the performance and capabilities of the model depending on the specific architecture and task requirements.

#### Convolutional neural networks

Convolutional neural networks are deep learning models that achieve successful results especially in tasks such as image processing, computer vision and object recognition. CNNs consist of multiple successive layers where the output of one layer is the input of another layer and are based on the feedforward working principle. These models use filters between layers to extract data-specific feature maps (Cai et al. 2023). Convolution, pooling and fully connected layers allow automatic learning of important features in the data. These models, which have high accuracy rates especially in big and complex datasets, require high computational power during the training process if the number of parameters is too high. The risk of overfitting is another challenge to consider. Correct configuration of the model, selection of appropriate hyperparameters and optimization strategies according to data size positively affect the success of CNNs.

#### Long short-term memory

Long short-term memory networks are deep learning architecture widely used in time series data and sequential data structures. On the other hand, RNNs are models that contain directed loops in memory and show superior performance especially in sequential data. Unlike traditional neural networks and RNNs, LSTM is better at remembering and forgetting data over time, which makes it preferred in tasks where sequential data is critical, such as language modeling, audio processing, and financial forecasting (Van Houdt et al. 2020). The basic component of LSTM unit is a memory cell that maintains an internal state that can be updated and changed over time. The memory cell stores information from previous time steps and selectively updates it based on the current input and its own internal dynamics. LSTMs use special structures called gates to control the flow of information (incoming and outgoing to the memory cell). There are three main types of gates in an LSTM unit: forget gate, input gate, and output gate. The forget gate determines what information from the previous cell state should be discarded or forgotten. The input gate determines what new information from the current input should be added to the cell state. The output gate determines what information from the cell state should be output to the next time step. The complex structures of these models require more computational power and training data due to the large number of parameters they contain.

#### Gated recurrent unit

GRU is a deep learning model that simplifies the LSTM architecture by combining input and forget gates into a single update gate. Like conventional LSTMs, GRUs have recurrent connections that allow information to persist across time steps in sequential data. At each time step, a GRU maintains a hidden state vector that captures information from previous time steps and acts as the network’s memory. This hidden state is dynamically updated when new input is processed. GRUs contain an update gate that controls the flow of information from the current input and the previous hidden state. The update gate decides how much of the previous hidden state should be preserved and how much of the new input should be integrated into the updated hidden state. In addition to the update gate, GRUs also include a reset gate. The reset gate determines how much of the past information should be forgotten or reset in the calculation of the new hidden state. GRU has the ability to learn long-term dependencies and is more computationally efficient than LSTM. GRU has less parameters compared to LSTM and is trained using backpropagation over time. During training, gradients are propagated over the entire input sequence and updates are made to the network parameters to minimize the loss function (Xu et al. 2018). Although its simplified structure is advantageous for some problems, performance may not be as strong as LSTM for very complex data relationships. GRUs have become quite popular in recent years due to their simplicity, efficiency, and competitive performance in a variety of sequence modeling tasks, including natural language processing, speech recognition, and time series analysis. They offer a more streamlined alternative to LSTMs while maintaining the ability to capture long-term dependencies in sequence data.

### Data preprocessing and feature extraction

Different mental disorders are treated with different methods. When psychiatric symptoms, cognitive symptoms and neurological symptoms of the underlying disease come together, it is quite difficult for physicians to make a differential diagnosis. The application of neuropsychological tests together with physical and neurological examination and psychiatric evaluation also helps the clinician in shaping the treatment. Feature observation, biological tests and imaging techniques are the methods used to make a diagnosis. Since the first symptoms of diseases are detected based on observations, biological tests are used in case the symptoms increase, become more frequent or diversify. Although the symptoms can be analyzed visually in the diagnosis of diseases in general, the number of biological tests that will provide direct confirmation is limited. These tests make it easier to interpret the symptoms of the disease. In particular, with the monitoring of neurological factors and MRI techniques, the symptoms are tried to be interpreted correctly and the diagnosis is made accordingly. In this study, it was tried to find features for the interpretation of the symptoms and the diagnosis of the disease directly with neurological findings.

Data normalization, a fundamental preprocessing technique in machine learning applications, plays an important role in standardizing and scaling the input features to improve model performance and convergence. While normalization is necessary to ensure consistent and effective model training, it is equally important to understand its impact on the evaluation metrics. The evaluation of signal processing and ML applications in the presence of statistical noise such as Gaussian noise is a different data preprocessing process. The Fourier transform is a fundamental mathematical tool used to analyze and represent functions or signals in terms of their frequency components. This transform decomposes a complex signal into a spectrum of simpler sinusoidal waves with varying frequencies and amplitudes. DWT performs multi-resolution analysis using high-pass and low-pass filters to extract detailed and approximate components of the signal. It is widely used in image processing, signal compression, and other applications where varying levels of detail are critical. Unlike the Fourier transform, which decomposes signals into sinusoids, DWT uses wavelets, which provide a more flexible representation, especially for non-stationary signals. CWT allows for the separation and analysis of signals by extending the concept of wavelets to continuous-time signals for signals of different scales and frequencies. Unlike the Fourier transform, which represents signals using sinusoids, CWT uses wavelets, which are oscillating functions with finite energy and localized support. The Burg method is a popular technique for spectral estimation in signal processing. It is known for its ability to produce high-resolution power spectral density estimates even with small datasets. This method is a spectral estimation technique that relies on autoregressive models to estimate the power spectral density of a signal. An autoregressive model assumes that a signal can be represented as a linear combination of previous values and white noise. Hyperparameter optimization, a part of ML model development, involves fine-tuning the parameters that govern the behavior and performance of the algorithms. Hyperparameter optimization tries to strike a balance between model complexity and performance, avoiding overfitting and seeking configurations that generalize well to the data. The choice of evaluation metrics in hyperparameter optimization has great impacts on model optimization and performance. While accuracy remains a common metric for classification tasks, other metrics such as precision, recall, F1-score, and receiver operating characteristic area under curve (ROC-AUC) offer more nuanced assessments of model performance, especially in imbalanced or asymmetrically cost-sensitive domains. In regression tasks, metrics such as mean square error (MSE), mean absolute error (MAE), and R-squared (R^2^) provide information on prediction accuracy and model fit.

### Evaluation metrics

Evaluation metrics help measure how well a system or model performs in performing its intended task. They also play important role in model selection, hyperparameter tuning, and performance optimization. In a binary classification task, data samples are typically predicted to be positive or negative. Each predicted binary label has four possible states: true positive (TP) is a correctly predicted positive result, true negative (TN) is a correctly predicted negative result, false positive (FP) is a positive predicted negative example, and false negative (FN) is a negative predicted positive example. Confusion matrix is a tabular representation of the model’s predictions against the true classes, facilitating detailed analysis of classification performance. The number of rows and columns of this matrix increases with the number of classes in the problem. The most commonly used evaluation metrics for binary classification are accuracy, sensitivity (or recall), specificity, and precision (Rainio et al. 2024). These refer to the percentage of correctly classified examples in the set of all examples, truly positive examples, truly negative examples, or examples classified as positive, respectively. In diagnostics, sensitivity or recall is also known as true positive rate, specificity as true negative rate, and precision as positive predictive value. Except for accuracy, the above-mentioned metrics are often used in pairs such as precision and recall or sensitivity and specificity. It is noteworthy that sensitivity and specificity reveal more information about the model than accuracy, especially when the number of true positive and negative examples is very unbalanced. The F1-core is a harmonic average of precision and recall. The equations for accuracy, sensitivity, specificity, precision and F1-score are given below, respectively.

1 $$Accuracy = \frac{TP + TN}{{TP + TN + FP + FN}} \in [0,1],$$

2 $$Precision = \frac{TP}{{TP + FP}} \in \left[ {0,1} \right],$$

3 $$Sensitivity = Recall = \frac{TP}{{TP + FN}} \in [0,1],$$

4 $$Specificity = \frac{TN}{{TN + FP}} \in [0,1],$$

5 $$F1\,Score = \frac{2*Precision*Sensitivity}{{Precision + Sensitivity}} \in [0,1]$$

Among regression metrics, the most commonly used are MAE, MSE, root mean square error (RMSE), and R^2^. For regression tasks, MAE calculates the mean absolute difference between the predicted values and the actual values. MSE calculates the mean squared error between the predicted and actual values. RMSE is the square root of MSE. It provides an interpretable measurement in the same unit as the target variable. R^2^ is a statistical measure of how close the data is to the fitted regression line. Learning curves are graphical representations that show how the proficiency of a task or process improves over time with experience and practice. Three outcomes are derived from learning curves, and these are overfitting, model-fitting, and underfitting. Overfitting is a condition that can be observed when the validation curve is higher than the training data. This indicates that the model has memorized the results and can only achieve success on that data. The overfitting problem can be prevented by reducing the variables, adding more data, or stopping early. Underfitting is observed when the validation curve and the training data are exactly the same or very close. This problem is observed when the data is very diverse and the model cannot learn. Model-fitting is seen when the validation curve has a slightly higher phase and a healthy difference from the training curve.

## Simulation results

The main purpose of this study is to evaluate the applicability of artificial intelligence methods to different types of mental disorders from a broad perspective. In this context, the simulations performed are designed specifically to show the performance differences of AI methods and to reveal the effects of certain data preprocessing techniques. Our study does not aim to make a direct clinical diagnosis. Additional validation studies on larger and more diverse datasets are required for clinical adaptations. Making artificial intelligence models more accessible and interpretable for clinicians will increase the applicability of these technologies in the field of health. In this study, certain mental disorders with different data formats such as questionnaires, EEG signals, text, and images were evaluated with various artificial intelligence models. In the subsections, the datasets used are first introduced, and then the simulation results are given. The experiments were executed within the Google Colaboratory Computational environment, leveraging a GPU-accelerated backend equipped with 8 GB of RAM and 27 GB of storage capacity. The library modules used for the experiments are keras, seaborn, sklearn, matplotlib, pandas, text hammer, nltk.

### Datasets

#### Online gaming anxiety data (Dataset 1)

The dataset consists of data collected as part of a survey conducted among gamers worldwide. The survey consists of various questions that psychologists asked 14,250 patients who are generally prone to anxiety, have social phobia, and have low or no life satisfaction. This dataset was created to measure players’ anxiety and depression levels, playing time, social interaction levels, performance anxiety, and even emotional reactions when losing or winning in a game. The original data was compiled by Marian Sauter and Dejan Draschkow (Agrawal 2020). The dataset consists of 55 columns corresponding to each question asked in the survey. Most of the columns correspond to different scoring criteria used in psychology, such as generalized anxiety disorder, satisfaction with life and social phobia inventory scores. There are a few general questions about where people were born, why they play online games, etc.

**Fig. 2:**
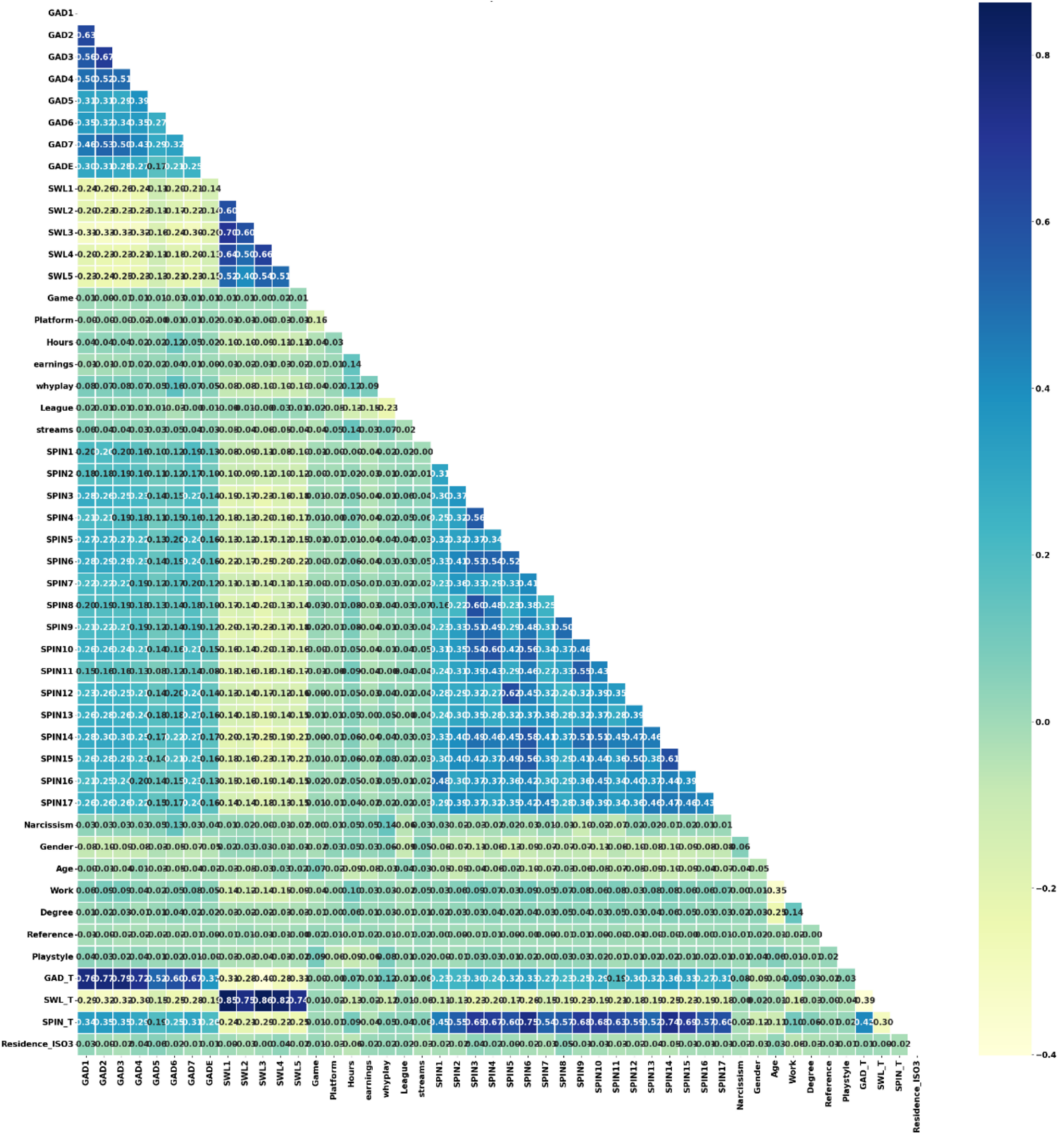
Correlation map of extracted features for Dataset 1

**Fig. 3:**
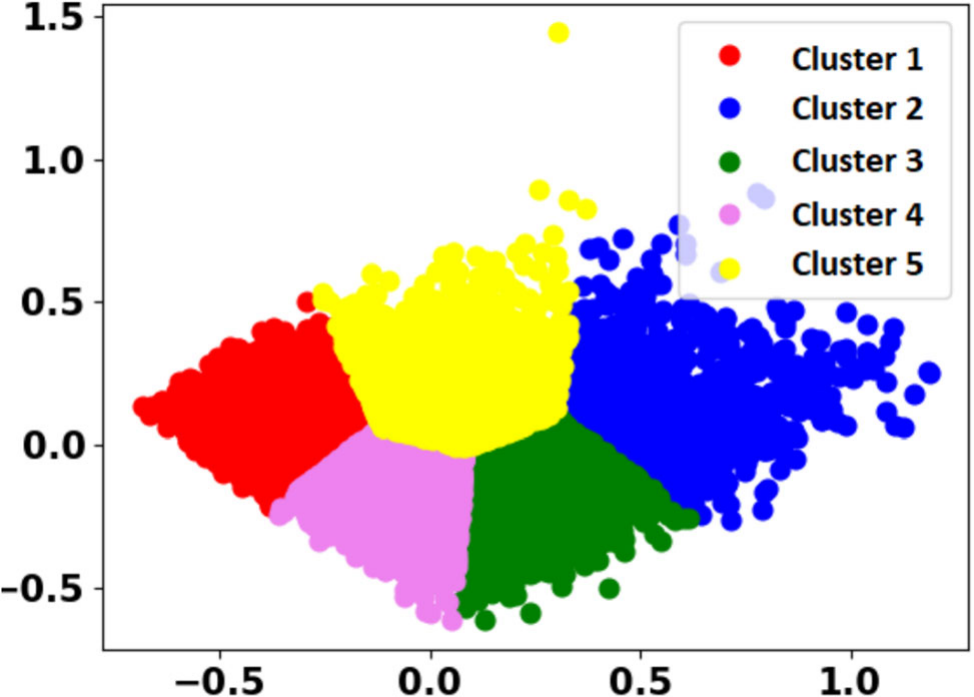
Clusters in Dataset 1

After the data review in the experimental studies, the “Hours” and “Streams” columns were combined and the information of the players who played more than 115 h and 0 h was removed from the dataset. Data standardization was performed for the League column. Birthplace and Birthplace_ISO3 columns were deleted. For most columns, missing or low values were filled with the most repeated value. Residence and Accept columns were removed. The labeled dataset was randomly divided into training and testing at a rate of 80%. After these arrangements in the dataset, 9664 of the total 12,081 data were separated for training and 2417 for testing. The correlation map showing the relationship between the data is given in Fig. 2. The dark areas in the correlation map are positive and indicate high correlation between the features. The light areas are negative and indicate low relationship between the features. 0.8 indicates a full correlation, 0.4 indicates a negative correlation, and 0 indicates no correlation at all. When we examine the map, it can be observed that there is a high correlation between the GAD_T value and the GAD1-GAD7 values; a high correlation between the SWL_T value and the SWL1-SWL5 values; and a high correlation between the SPIN_T value and the SPIN values. In the data normalization process, the MinMaxScaler function was used for the Hours, Streams, Age, GAD_T, SWL_T and SPIN_T columns. Clustering was performed for 5 different groups using the GAD_T, SWL_T, SPIN_T, Hours and streams columns and using the correlation map. The 5 different clusters (L0–L4) belonging to Dataset 1 are given in Fig. 3. When the relationships with the GAD_T, SWL_T, SPIN_T, Hours and Streams columns are examined one by one according to the labels, the following items are interpreted.

- L0: Prefers to play games rather than watch and is happy with life but has high social phobia.
- L1: Happy with life and there is no problem in playing alone or with people, that is, he/she does not have social phobia.
- L2: Happy with life but has high social phobia.
- L3: He/she has high anxiety and therefore is not happy with life at all.
- L4: He/she is not very happy with life, has anxiety and also has social phobia.

In the simulation studies, the number of trees is 200, the depth value is 200, the learning coefficient is 0.1, the row ratio is 0.7 and the column subsampling value is 0.8 for the XGBoost model. The model parameters are used with their default values in the LightGBM algorithm. The value of the column subsampling parameter is 1.0, the learning coefficient is 0.1, the minimum sample weight is 20 and the number of leaves is 3. The number of neighbors is selected as 3 for the KNN algorithm and the other parameters are used as default. The number of trees is 1000, the random number generator is 44 and the tree depth is 8 for the RF algorithm. The other parameters are default values. The default parameter values of the model are used for the SVM algorithm. The performance results of all artificial intelligence models used in this study for Dataset 1 are listed in Table 1.

**Table 1:** The classification results of the machine learning models used for Dataset 1

Cluster	Metrics	XGBoost	LightGBM	KNN	RF	SVM
L0	Precision	0.94	0.98	0.60	0.81	0.73
	Sensitivity	0.93	0.98	0.57	0.80	0.73
	F1-score	0.93	0.98	0.59	0.80	0.73
L1	Precision	0.96	0.93	0.86	0.89	0.88
	Sensitivity	0.97	0.98	0.69	0.86	0.82
	F1-score	0.97	0.96	0.77	0.87	0.85
L2	Precision	0.94	0.96	0.41	0.72	0.62
	Sensitivity	0.89	0.94	0.62	0.77	0.72
	F1-score	0.91	0.95	0.49	0.75	0.67
L3	Precision	0.90	0.96	0.53	0.76	0.67
	Sensitivity	0.96	0.97	0.85	0.96	0.91
	F1-score	0.93	0.96	0.65	0.85	0.77
L4	Precision	0.95	0.94	0.65	0.83	0.79
	Sensitivity	0.95	0.92	0.61	0.78	0.71
	F1-score	0.95	0.93	0.63	0.81	0.75

Then, hyperparameter optimization is performed using the grid search method. In the XGBoost model, the best results in the grid search result were determined as 1 for the column subsampling value, 0.09 for the learning coefficient, 4 for the tree depth, 250 for the number of trees, and 0.6 for the row ratio. In the LightGBM algorithm, the most successful parameter results with hyperparameter optimization were calculated as 0.1 for the learning coefficient, 4 for the tree depth, 150 for the minimum number of data in the leaf, 220 for the tree number value, 1200 for the leaf number value, and 0.1 for the row ratio value. For the KNN algorithm, the most suitable neighbor number parameter was determined as 20 with hyperparameter optimization. When hyperparameter optimization was performed with the random search algorithm instead of the grid search algorithm in the RF algorithm, the best parameters were calculated as 800 for the number of trees, 6 for the minimum number of samples, 1 for the minimum number of leaves, 3 for the number of features, and 110 for the maximum depth. The parameters selected by hyperparameter optimization for SVM are 10 for C and 0.1 for the node score. The kernel gave linear results. The results of the repeated experiments with the most suitable parameters for all artificial intelligence models are given in Table 2.

**Table 2:** The classification results of the machine learning models with hyperparameter optimization for Dataset 1

Cluster	Metrics	XGBoost	LightGBM	KNN	RF	SVM
L0	Precision	0.98	0.98	0.95	0.94	0.98
	Sensitivity	0.98	0.98	0.74	0.87	0.98
	F1-score	0.98	0.98	0.83	0.91	0.98
L1	Precision	0.94	0.94	0.58	0.82	0.94
	Sensitivity	0.97	0.98	0.92	0.88	0.96
	F1-score	0.96	0.96	0.71	0.85	0.95
L2	Precision	0.96	0.98	0.62	0.83	0.95
	Sensitivity	0.96	0.94	0.68	0.82	0.96
	F1-score	0.96	0.96	0.65	0.83	0.96
L3	Precision	0.96	0.95	0.80	0.86	0.97
	Sensitivity	0.97	0.98	0.67	0.87	0.97
	F1-score	0.97	0.97	0.73	0.86	0.97
L4	Precision	0.96	0.95	0.39	0.86	0.97
	Sensitivity	0.92	0.92	0.76	0.93	0.95
	F1-score	0.94	0.93	0.52	0.89	0.96

In the simulation studies conducted before hyperparameter optimization for the first dataset, the most successful result was obtained with LightGBM. After hyperparameter optimization, the biggest change was in SVM with a performance increase from 0.76 to 0.97 as shown in Table 3. When the performance of different AI models on “Online Gaming Anxiety Data” was evaluated, it was seen that hyperparameter optimization led to significant improvements in the accuracy, sensitivity and F1-score values of the models. SVM was the model with the largest improvement after optimization and is now competitive with LightGBM. KNN showed significant improvements in some clusters (Cluster 0 and 1), but overall it was considered a weak or unstable model for this dataset. Although RF showed improvement with optimization, it fell slightly behind XGBoost, LightGBM and SVM. Overall, hyperparameter optimization significantly increased the performance of all models.

**Table 3:** The performance comparison with all methods for Dataset 1

Model	Accuracy
XGBoost	0.96
LightGBM	0.96
KNN	0.72
Random forest	0.87
SVM	0.97

#### Schizophrenia disorder EEG dataset (Dataset 2)

This dataset includes open access EEG signals obtained from 14 schizophrenia patients and 14 healthy controls. EEG signals were recorded under resting conditions with eyes closed for 15 min. Data were obtained with 19 EEG channels using the standard 10–20 EEG montage with a sampling frequency of 250 Hz. These channels are Fp1, Fp2, F7, F3, Fz, F4, F8, T3, C3, Cz, C4, T4, T5, P3, Pz, P4, T6, O1, O2. The signals of each EEG channel were filtered using a 2nd order Butterworth filter in the following physiological frequency bands: 2–4 Hz (delta), 4.5–7.5 Hz (theta), 8–12.5 Hz (alpha), 13–30 Hz (beta), 30–45 Hz (gamma). The patient group consisted of seven males (mean age 27.9 ± 3.3 years) and seven females (mean age 28.3 ± 4.1 years) diagnosed with paranoid schizophrenia according to the International Classification of Diseases (ICD)-10-CM criteria (F20.0) and showing significant positive symptoms (Olejarczyk and Jernajczyk 2017). Inclusion criteria for the original study were: a minimum age of 18, ICD-10 diagnosis F20.0, and medication washout period of a minimum of 7 days. In the data preprocessing process, channel references of edf files were first calculated and the calculated reference value was subtracted from each channel to reduce noise. In simulations, new sequences were created for feature extraction from EEG data using Fourier transform, DWT, CWT and Burg methods, respectively. Then, CNN, LSTM and GRU models were designed. In order to obtain the best result for each model, the number of layers and model parameters were changed and the most appropriate architecture was decided. The models given here are the models with the best results.

In the simulation studies, a 1D convolution layer was designed in the first two layers for the CNN model. The number of filters was selected as 16, the number of kernels was selected as 2, and ReLU was used as the activation function. 1D pooling layer was selected as the third layer. A drop-out layer was used in the fourth layer to prevent memorization. The same sequence was repeated for the next 4 layers and the number of filters was increased to 32. Since there will be two category selections in the last layer of the model, the sigmoid function was used as the activation function. The learning rate of the model was selected as 0.01. In order to prevent overfitting during the training phase of the model, the control point values of the model were recorded. The number of steps was selected as 100. The accuracy rate of the model was 0.81. The label 0 represents the healthy control group, and the label 1 represents the schizophrenia label. In the sensitivity metric, the data with the label schizophrenia gave a more successful result at a rate of 0.88. In the sensitivity metric, the control group showed a higher success with a rate of 0.91. The control group again showed a higher success for the F1-score. In the simulation studies, 1D time-distributed convolution layer was selected for the first three layers of another artificial intelligence model designed, LSTM. The number of filters was determined as 16, the number of kernels was determined as 2, and ReLU was used as the activation function. A dropout layer was used in the fourth layer to prevent overfitting. A time-distributed pooling layer was used in the fifth layer. A time-distributed smoothing layer was used in the sixth layer. LSTM layers were used in the seventh and eighth layers. A dropout layer was used again in the ninth layer. A smoothing layer was used in the tenth layer. Since there was a binary category in the output layer, a sigmoid activation function was used. The number of training steps of the model was selected as 100, and the learning rate was selected as 0.01. The training accuracy rate was 0.83. The sensitivity rate of schizophrenia disorder was obtained as 0.86, which was higher than the control group. For the sensitivity metric, the control group gave a higher result with a rate of 0.86. Both groups gave equal results for the F1-score. For the GRU model designed for the EEG dataset belonging to SD, a 600-unit GRU layer was used in the first layer. A dropout layer was used in the second layer. A 300-unit GRU layer was used in the third layer. A dropout layer was used in the fourth layer to prevent excessive overfitting. A flattening layer was used in the fifth layer. After the intermediate layers, sigmoid activation was used in the last layer. As a result of the training, the accuracy rate was 0.82. It gave equal results in sensitivity, precision and F1-score rates for all categories. The performance comparison of all artificial intelligence models designed for the second dataset is presented in Tables 4 and 5. The most successful result in the accuracy rate comparison of the models is seen in the LSTM and GRU models.

**Table 4:** The classification results of the deep learning models with hyperparameter optimization for Dataset 2

Cluster	Method	Precision	Sensitivity	F1-score
0 (normal)	CNN	0.74	0.94	0.83
	LSTM	0.78	0.89	0.83
	GRU	0.80	0.86	0.83
1 (schizophrenia)	CNN	0.92	0.68	0.78
	LSTM	0.88	0.76	0.82
	GRU	0.86	0.79	0.82

**Table 5:** The performance comparison with all methods for Dataset 2

Model	Accuracy
CNN	0.81
LSTM	0.83
GRU	0.82

In this study, in order to examine the performance of artificial intelligence models in distinguishing schizophrenia patients and healthy individuals from open access EEG signals, the performance of CNN, LSTM and GRU models was analyzed by performing feature extraction on the data with Fourier transform, DWT, CWT and Burg methods. According to the performance results obtained after hyperparameter optimization presented in Tables 4 and 5, the most successful results among CNN, LSTM and GRU models were observed in LSTM and GRU models. While the CNN model showed higher sensitivity in some clusters, LSTM and GRU models performed better in terms of overall accuracy.

#### Autism spectrum disorder datasets (Dataset 3)

In this study, 3 datasets were combined to examine autism spectrum disorder. The first of these datasets consists of 1986 records and includes factors that play a role in the development of ASD in children (A10 ASD Section, Social Responsiveness Scale, Year of Age, Qchat_10_Score, Speech Delay/Language Disorder, Learning Disability, Genetic Disorders, Depression, Global Developmental Delay/Intellectual Disability, Social/Behavioral Problems, Childhood Autism Rating Scale, AB, Gender, Ethnicity, Jaundice, ASD Family Memory, etc.). The second dataset was developed by Dr. Fadi Thabtah using a mobile application called ASDTests to screen for autism in young children (Thabtah 2017). In this dataset, ten behavioral characteristics (Q-Chat-10) and other individual characteristics that have been proven to be effective in detecting ASD cases from behavioral controls were recorded. The number of records in the dataset is 1054 and the number of class variables is 18. The third dataset consists of survey results from over 700 adolescents and adults who filled out the application form. Labels are available indicating whether the person has been diagnosed with autism, allowing ML models to predict the probability of autism, allowing health professionals to prioritize their resources (Thabtah 2017). In order to use the dataset in the experiments, similar columns and features of the three datasets were determined and a new dataset consisting of 3473 records was created. Similar columns for the three datasets are A1–A10, Age, Gender, Ethnicity, Autism Diagnosis in the Family, Test Completer and ASD Diagnosis. For the data belonging to individuals consisting of 4 groups as children aged 0–10, adolescents aged 11–15, youth aged 16–24 and adults over 25, the label 0 represents the control group and the label 1 represents the group diagnosed with autism. Then, data normalization was performed.

In the simulations, the number of trees for the XGBoost model was given as 200, the maximum depth value as 7, the learning coefficient as 0.1, the row ratio as 0.7 and the column subsampling value as 0.8. The simulations were performed for dataset 3 using the default parameters of the algorithm for LightGBM, KNN and SVM from the machine learning algorithms. Among the parameters of the RF algorithm, the tree depth value was selected as 10, the random number generator was selected as 0, and the other parameters were used with their default values. The performance results of all artificial intelligence models used in this study for Dataset 3 are listed in Table 6. Then, hyperparameter optimization was performed using the grid search method. In the XGBoost model, the number of trees was obtained as 200, the tree depth value as 8, the learning coefficient as 0.1, the row ratio as 0.7, and the column subsampling value as 0.8 as a result of the grid search. As a result of the hyperparameter optimization, the learning coefficient was calculated as 0.8, the tree depth as − 2, the minimum value in the leaf as 150, the number of trees as 130, the number of leaves as 150, and the row ratio as 0.9 from the LightGBM parameters. In KNN, hyperparameter optimization was applied only for the neighbor number parameter, and the best value of the neighbor number, whose value range was between 1 and 31, was calculated as 5. In order to find the best parameters of the RF method with hyperparameter optimization, the random search algorithm was used and the number of trees was obtained as 300, the minimum number of samples as 6, the minimum number of samples in the leaf as 1, the number of features as 2, and the tree depth as 90. The parameters selected using the grid search algorithm with hyperparameter optimization for SVM yielded results as 10 for C, 0.1 for the node score, and the kernel function as poly. The repeated experiment results with the most suitable parameters of all artificial intelligence models for Dataset 3 are presented in Table 7.

**Table 6:** The classification results of the machine learning models used for Dataset 3

Cluster	Method	Precision	Sensitivity	F1-score
0 (control)	XGBoost	0.97	0.99	0.98
	LightGBM	0.97	0.99	0.98
	KNN	0.89	0.93	0.91
	RF	0.96	0.98	0.97
	SVM	0.88	0.80	0.84
1 (diagnosed with autism)	XGBoost	1	0.98	0.99
	LightGBM	0.99	0.97	0.98
	KNN	0.94	0.91	0.93
	RF	0.98	0.97	0.98
	SVM	0.82	0.89	0.85

**Table 7:** The classification results of the machine learning models with hyperparameter optimization for Dataset 3

Cluster	Method	Precision	Sensitivity	F1-score
0 (control)	XGBoost	0.97	0.99	0.98
	LightGBM	0.98	0.97	0.98
	KNN	0.89	0.93	0.91
	RF	0.96	0.99	0.97
	SVM	0.95	0.98	0.97
1 (diagnosed with autism)	XGBoost	1	0.98	0.99
	LightGBM	0.98	0.98	0.98
	KNN	0.94	0.91	0.93
	RF	0.99	0.97	0.98
	SVM	0.98	0.96	0.97

The performance of different machine learning models for ASD classification was measured by combining three datasets. As a result of simulation studies, it was seen that XGBoost and LightGBM algorithms achieved the highest accuracy rates, and the RF model achieved a similar success as seen in Table 8. In addition, an increase in the success of all models was observed with hyperparameter optimization. XGBoost and RF models achieved the highest performance after optimization and were determined to be the most suitable models for the detection of autism spectrum disorder. The SVM model also increased its accuracy rate after hyperparameter optimization and became competitive with other algorithms.

**Table 8:** The performance comparison with all methods for Dataset 3

Model	Accuracy
XGBoost	0.98
LightGBM	0.98
KNN	0.92
Random forest	0.98
SVM	0.97

#### Suicide and depression detection dataset (Dataset 4)

The Suicide and Depression Detection dataset is designed to support the development of machine learning models aimed at detecting suicidal thoughts and depression in text-based data. The data collected using the Reddit platform’s Pushshift API is a collection of posts from the “SuicideWatch”, “depression”, and “teenagers” subreddits. Posts from the “SuicideWatch” (December 16, 2008–January 2, 2021) subreddits are labeled as suicide, while posts from the “depression” (January 1, 2009–January 2, 2021) subreddits are labeled as depression. Posts from the “teenagers” subreddit serve as examples of normal conversations, providing non-suicide content for comparison. Several different versions of the dataset are available: a simplified version that includes only suicide and non-suicide labels, and a more detailed version with three labels (suicide, depression and normal) (Komati 2021). The dataset consists of two columns specifying the post content and depression/suicide categorization for sentiment analysis. The texts were divided into tokens. Then, a list of words that did not affect the psychological state was created and these were removed from the dataset. All words were converted to lower case, e-mail information, punctuation marks and special letters were removed. Then, 10,000 unique words were selected and the dataset was randomly divided into training and testing with a ratio of 0.3.

In the simulation studies, the embedding layer was used in the first layer for the CNN model. In this layer, the number of words for the input size (10,000) and the embedding size (100) were used for the output size. The 1D convolution layer was used as the second layer. The number of filters was 16, the number of kernels was 5, and ReLU was used as the activation function. 1D pooling was selected as the third layer. The fourth, fifth, sixth, and seventh layers are the repetition of the second and third layers. A flattening layer was used in the eighth layer. A dense layer was used in layers 9–11. The eleventh layer is also the output layer and the softmax activation function was used as the activation layer. The number of steps of the CNN model was selected as 25. In the training, the checkpoint values were recorded to prevent overfitting and the training was stopped in the 11th step. In the training process, the label 0 represents those who do not have suicidal tendencies and the label 1 represents those who have suicidal tendencies. There is an embedded layer in the first layer of the LSTM model designed for Dataset 4. The input layer size is the same as the number of words. The drop layer was used in the second layer. The LSTM layer was used with 32 filters in the third layer. The drop layer was used to prevent memorization in the fourth layer. The pooling layer was used in the fifth layer. The last layer is the output layer and softmax was used as the activation function. For the GRU model, the embedded layer was used in the first layer, the drop layer in the second layer, the GRU layer with 100 units in the third layer, the drop layer with a ratio of 0.2 in the fourth layer, and the GRU with 100 units in the fifth layer. The softmax activation function was used in the last layer. The performance comparison of all artificial intelligence models designed for Dataset 4 is presented in Table 9. The most successful result in the comparison of the accuracy rate of the models belongs to the GRU model, as seen in Table 10.

**Table 9:** The classification results of the deep learning models with hyperparameter optimization for Dataset 4

Cluster	Method	Precision	Sensitivity	F1-score
0 (normal)	CNN	0.92	0.89	0.90
	LSTM	0.91	0.88	0.89
	GRU	0.94	0.91	0.93
1 (depression and suicidal tendencies)	CNN	0.89	0.92	0.91
	LSTM	0.88	0.91	0.90
	GRU	0.91	0.95	0.93

**Table 10:** The performance comparison with all methods for Dataset 4

Model	Accuracy
CNN	0.90
LSTM	0.90
GRU	0.93

In this study, three different deep learning models designed for the Suicide and Depression Detection dataset were used to detect depression and suicidal tendencies. According to the simulation results, GRU performed better than CNN and LSTM models in detecting both suicidal tendencies and depression symptoms. The results of the CNN and LSTM models are quite close to each other. These results show that each model has different advantages in certain scenarios. However, in general, it was determined that the GRU model was the most effective model with higher success rates in text-based depression and suicide detection applications.

#### Dementia MRI dataset (Dataset 5)

**Fig. 4:**
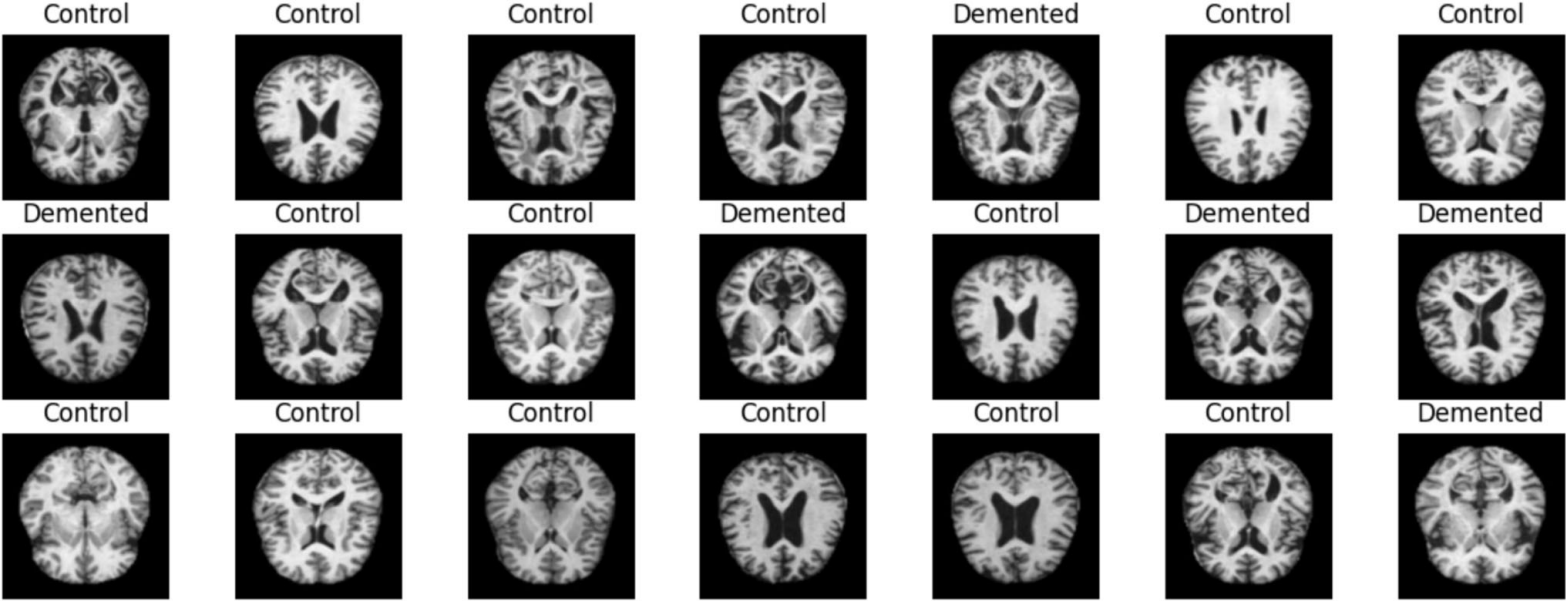
Example MRI images labeled with and without dementia in Dataset 5

The data in the dementia MRI dataset was collected from various websites/hospitals/public repositories (Mahmud et al. 2024). The dataset contains 6400 pre-processed MRI images sized as 128 × 128 pixels. According to the degree of dementia, the dataset contains 896 mild dementia, 64 moderate dementia, 3200 non-dementia and 2240 very mild dementia MRI images. In this study, a total of 3200 data were labeled as dementia by combining mild dementia, moderate dementia and very mild dementia images. Data without dementia were also used by labeling them as non-dementia. The images were rescaled to 1/255 and data normalization was performed. An example data visual is presented in Fig. 4.

In the simulation studies, the input layer for the CNN model was used as the normalization layer and the Gaussian noise in the data was removed. The 2D convolutional layer was used as the second layer. The number of filters was selected as 32, the kernel size as a 3 × 3 matrix and the activation function as ReLU. A batch normalization layer was used in the third layer. A pooling layer in a 2 × 2 matrix was used in the fourth layer. The second, third and fourth layers were used by repeating them 3 more times. A smoothing layer was used in the fourteenth layer. The softmax activation function was used in the output layer. In the tests performed, it was seen that the accuracy value increased as the number of steps increased for both the training data and the validation data. The number of steps during the training of the model was selected as 40. The accuracy rate of this CNN model designed for the dataset with 2 classes as dementia and non-dementia was obtained as 0.98. The input layer for the LSTM model designed for this dataset was used as the normalization layer and the Gaussian noise in the data was removed. In the second and third layers, a 1D time-distributed convolution layer was selected. In both layers, the number of filters was 16, the number of kernels was 2, and the activation function was used as ReLU. In the fourth layer, a dropout layer with a ratio of 0.2 was used, in the fifth and sixth layers, time-distributed pooling and smoothing layers were used, and in the seventh layer, an LSTM layer was used. The softmax activation function was used in the output layer. During the training process, the number of steps was selected as 40, but in order to prevent overfitting, the control point values of the model were recorded. Accordingly, an accuracy value of 0.99 was obtained with the LSTM model for the 2-class dementia dataset. The input layer was used as the normalization layer for the GRU model and denoising was applied to the data. In the second layer, a rescaling, reshape layer was used to prepare the data size for the GRU layer. In the third layer, a 600-unit GRU, in the fourth layer a dropout layer, in the fifth layer a 300-unit GRU layer, in the sixth layer a dropout layer, and in the seventh layer a smoothing layer were used. The softmax activation function was used in the last layer. In the simulation studies, the accuracy rate of the GRU model was obtained as 0.99. The performance comparison of all artificial intelligence models designed for the dementia MRI dataset is presented in Table 11. CNN is available in all deep learning models designed for the dementia dataset. The comparison of the accuracy values of the models is listed in Table 12. Accordingly, the most successful models are the LSTM and GRU models with an accuracy rate of 0.99. According to the learning curves, the less fluctuation in the learning phase of the LSTM model shows that it is more reliable than the GRU model.

**Table 11:** The classification results of the deep learning models with hyperparameter optimization for Dataset 5

Cluster	Method	Precision	Sensitivity	F1-score
0 (non-dementia)	CNN	0.98	0.98	0.98
	LSTM	0.99	0.99	0.99
	GRU	0.98	0.99	0.99
1 (dementia)	CNN	0.98	0.98	0.98
	LSTM	0.99	0.99	0.99
	GRU	0.99	0.98	0.99

**Table 12:** The performance comparison with all methods for Dataset 5

Model	Accuracy
CNN	0.98
LSTM	0.99
GRU	0.99

This study aims to detect mental disorders using various AI approaches from different types of data sources such as surveys, EEG signals, text and images. Simulation studies include predicting anxiety and depression levels in online games, detecting schizophrenia from EEG signals, detecting autism spectrum disorder, analyzing text-based indicators of suicidality and depression, and diagnosing dementia from brain MRI images. Table 13 provides a comparative summary table of the advantages and limitations of the machine learning and deep learning models used in this study in terms of diagnosing different mental disorders. The computational costs of the AI models used refer to the processing power required to train the model, the validation process, and make predictions. Such a cost varies depending on the size of the dataset used, the complexity of the model and the processing time. Considering factors such as CPU or GPU requirements and memory usage for real-time applications, Table 13 also evaluates the computational costs of each model used in this study.

**Table 13:** Comparative summary table explaining the advantages and limitations of AI models used in this study in diagnosing different disorders

Model	Datasets	Advantages	Limitations	Computational cost
XGBoost	Dataset 1 (anxiety) Dataset 3 (ASD)	High accuracy Good generalization ability Fast training time Does not require much parameter tuning Efficient on big datasets	Performance loss on big feature sets May require many hyperparameters Long training times on big datasets	Medium (fast training time, low memory usage)
LightGBM	Dataset 1 (anxiety) Dataset 3 (ASD)	High speed on big datasets Low memory usage Requires less parameter tuning Better performance on big datasets	Risk of overfitting on small datasets Limited in learning more complex structures Less effective on time series data	Low (fast, low CPU/GPU requirements)
KNN	Dataset 1 (anxiety) Dataset 3 (ASD)	Simple and understandable Less data preprocessing required	Slow estimation on big datasets Requires a lot of data for fast estimations High memory usage due to data in training	High (high memory and processing power requirements depending on data size)
RF	Dataset 1 (anxiety) Dataset 3 (ASD)	Does not require much parameter tuning Resistant to overfitting Gives the importance of features so it is good for data that requires feature selection	Training time can be long Large number of trees increases processing time	Medium (may take more time to train, medium memory requirement)
SVM	Dataset 1 (anxiety) Dataset 3 (ASD)	High accuracy in small datasets Strong generalization ability Successful in high-dimensional data	Training time can be very long on big datasets Complex kernel functions are time consuming	Medium (computational load increases for very big datasets and complex kernel functions)
CNN	Dataset 2 (schizophrenia EEG) Dataset 4 (depression and Suicide tendencies) Dataset 5 (dementia MRI)	More successful in image and EEG data Provides more powerful feature extraction with deep learning methods	High processing power and memory usage Long training time Performance may be limited on time series data	High (large number of parameters, high CPU/GPU requirements)
LSTM	Dataset 2 (schizophrenia EEG) Dataset 4 (depression and suicide tendencies) Dataset 5 (dementia MRI)	Strong in modeling time series and long dependencies Long-term dependencies can be learned with less information loss Suitable for EEG and text based data	Requires high computing power Long training time	Very high (high computational load to handle long-term dependencies)
GRU	Dataset 2 (schizophrenia EEG) Dataset 4 (depression and suicide tendencies) Dataset 5 (dementia MRI)	Provides faster training than LSTM Has the ability to learn short-term dependencies	May be limited in learning more complex dependencies Risk of overfitting on small datasets May fail if learning speed is not sufficient	Medium (less computational effort compared to LSTM)

## Discussion and conclusion

The detection of mental disorders with artificial intelligence methods has shown significant development in recent years. These algorithms can identify the symptoms of mental disorders in the early stages by analyzing individuals’ speech, behavior or daily activities before obvious symptoms appear. Early detection of disorders such as anxiety, depression, autism spectrum disorder, suicidal tendencies, schizophrenia and dementia in particular allows the treatment process to be accelerated and managed more effectively. Since mental disorders are usually affected by multiple factors, it is difficult to diagnose such disorders correctly. In order to perform data analysis correctly, it is first necessary to obtain the correct data. Artificial intelligence methods such as machine learning or deep learning models are extremely effective in detecting complex patterns in big datasets. This data used to detect emotional and psychological states may consist of individuals' social media posts, daily activities, biometric data (facial expression, tone of voice, heart rate, eye movement, etc.) or other digital traces. On the other hand, neurological disorders such as schizophrenia and dementia are generally associated with changes in brain functions. Artificial intelligence methods can monitor such disorders by analyzing brain imaging data (such as MRI, CT, PET scans). From the data obtained with imaging techniques, structural and functional changes in the brain can be identified so that the treatment process can be planned more accurately and effectively.

In this study, a comprehensive literature review was conducted including various AI models used for mental disorders due to the many beneficial features mentioned above. Then, specific mental disorders that are openly accessible with different data formats such as survey, EEG signal, text or image were determined. In this context, critical issues such as estimating anxiety and depression levels of individuals playing online games, detecting schizophrenia from EEG signals, determining autism spectrum disorder with machine learning, text-based suicide and depression detection and dementia diagnosis from brain MRI images were addressed. In the first stage of the simulation studies, a comprehensive data preparation process was carried out to make the datasets suitable for modeling processes. This process included steps such as cleaning the data, removing unnecessary data, wavelet transforms and data normalization. These steps facilitated the learning of the model and made the data more usable. In particular, correcting data that had the same meaning with different spellings also contributed positively to the learning process of the model. In addition, clustering techniques supported classification decisions in datasets that were difficult to label, and wavelet transforms were used to increase the usability of the data for data containing signals. The most appropriate models were designed for these datasets with different data formats, and the classification results obtained were compared with the results obtained using optimized model parameters. In estimating anxiety and depression levels, LightGBM and optimized SVM models achieved the most successful results with 96% sensitivity and F1-score. In detecting schizophrenia patients from EEG signals, LSTM and GRU models exhibited superior performance with 83% and 82% accuracy rates. In detecting autism spectrum disorder, XGBoost and LightGBM algorithms reached 98% accuracy rate, while the optimized SVM model also reached a competitive level. In text-based suicide and depression detection, the GRU model showed the best performance with 93% accuracy rate, while CNN and LSTM models also gave successful results at 90% levels. In dementia detection, LSTM and GRU models reached 99% accuracy rate, and the CNN model reached 98% accuracy rate. When all datasets and all models were evaluated, the most successful results were obtained with LightGBM, optimized SVM, LSTM and GRU models. In particular, the superior performance of LSTM and GRU models on time-dependent data is remarkable and these models can be considered as strong alternatives in the fields of medical imaging and natural language processing. In addition, powerful machine learning algorithms such as XGBoost and LightGBM have also proven to be effective tools with high accuracy rates in clinical diagnosis processes. The analyses performed showed that hyperparameter optimization significantly increased the performance of the models and differentiated the most effective model selection for certain problems. In addition, while working with these models, data preprocessing and feature selection processes made significant contributions to optimizing model performances.

The artificial intelligence approaches used in this study offer significant advantages for both the analysis of large-scale datasets and individual symptom detection. Based on the numerical results obtained, tree-based methods such as XGBoost, LightGBM and RF, which provide high accuracy, can be preferred for larger-scale data analyses compared to datasets collected for disorders examined at the individual level. Such models are more effective in determining common disorder models or risk factors with a large number of patient data. On the other hand, individual diagnosis and symptom monitoring require different approaches. It is known that models based on sequential data analysis such as LSTM and GRU are more effective in monitoring the health status of individuals over time and in personalized diagnosis processes. Especially in time-dependent data such as EEG signals or speech data, the preference of such models helps to determine individual symptom patterns. This contributes to early diagnosis processes. In addition, shallow AI approaches such as SVM and KNN also yield successful results with smaller datasets. In this sense, when these methods are preferred for individual patient evaluations, fast and effective results can be obtained without requiring high computational costs. These differences between large-scale analysis and individual diagnostic methods broaden the applicability of AI-based healthcare solutions. While large-scale data analysis is critical for general diagnostic protocols and population-level risk prediction, personalized approaches help in more accurate and sensitive diagnostic processes on an individual basis.

Different data sources, sample diversity in these datasets, potential ethical issues, clinical generalizability, and limitations in decision-making in a clinical setting are critical to the reliability of AI-based diagnostic systems. In this sense, in particular, an individual symptom or a specific anomaly may not definitively indicate a mental disorder. In the clinical diagnosis process, multiple symptoms and medical history should be evaluated together. AI models help clinical evaluation by identifying risk factors in individuals. Such analyses alone may not be sufficient for definitive diagnosis in individual symptom patterns at the moment. Considering ethical concerns, the use of AI-based systems together with clinical experts as a preliminary screening or supportive tool is more common.

In conclusion, this study has demonstrated the potential of artificial intelligence techniques for the detection of mental disorders and has shown how effective various artificial intelligence models can be on different types of data. Artificial intelligence-based clinical decision support systems can enable faster and more accurate diagnosis of patients, allowing for early intervention and helping to implement personalized treatment plans. At the same time, these systems can support the individual development of individuals with mental disorders by offering rehabilitation programs according to their specific needs. Future studies can generalize the findings of this study with larger sample groups and different data sources. Transfer learning or hybrid use of different artificial intelligence models can make clinical decision support systems more sensitive and reliable. In addition, testing the obtained models in a clinical environment and evaluating their applicability can further strengthen the role of artificial intelligence in the field of health.

## Data Availability

No datasets were generated or analysed during the current study.
